# A step in the right direction, towards a feminist policy analysis

**DOI:** 10.3402/gha.v6i0.22818

**Published:** 2013-09-20

**Authors:** Carlos Alvarez-Dardet

**Affiliations:** CIBERESP, Center for Research in Epidemiology and Public Health, University of Alicante, Spain

A century after the foundation of the feminist movement (1) and 40 years after the beginning of gender studies in the academic world, effective gender equity (2) remains as an elusive objective on the political horizon.

One of the possible reasons for this unfair situation is the lack of scientific research in identifying entry points and policy levers for effective and coherent changes in politics, policy, and governance. In other words, change is about simultaneous shifts in power, range of actions, and culture.

In other areas of social life, the discipline of policy analysis is covering this range of objectives asking: Why? Who? Whom?, and fuelling with relevant information the needed activism to cope with social problems. This is the tradition of ‘speaking truth to power’ initiated by Aaron Wildavsky (3).

The Thesis of Belen Cambronero, published as a cover story earlier this year, (4) can be considered under the same umbrella (trying to uncover the truth) but in the sphere of feminist policy analysis.

Her innovative work systematically explores gender bias in areas of human activity apparently far away from each other, like the marketing practices of the pharmaceutical industry (5), public communication of Spanish institutions (6), or the political debate in the Spanish parliament on abortion laws (7) – all finding discrimination against women in these areas.

She also observed the same pattern in different settings with different internal rules, from advertisements to parliament. These observations are only small pieces in the huge jigsaw of rebuilding more equitable societies.

The trans-disciplinary nature of Belen's training PhD from the Multidisciplinary Gender Studies PhD Programme at the University of Alicante, coming from a degree in public relations, and having a sociologist and a medical doctor as her supervisors, has given its fruits in this article. She tried successfully to tackle the social construction of reality through the analysis of communication relating to health issues using a wide range of methodological tool kits and traditions.

Her work is able to show gender biases in public and corporate policies, thus interrogating governments and political parties (from Spain) and companies (from the pharmaceutical industry) on the effect their policies have on women: it is an important step in the right direction and a very much needed construction of a real feminist policy analysis.

Interestingly, she came with very political conclusions, and I would like to highlight two of them:

1. Greater participation of women in public policy and decision making is critical for women's health, such as the issue of abortion, which is very much related to the MP's gender.
2. The need for a ‘caring institution’ she named as the ‘policy watch dog’ (maybe a less aggressive name should be selected to name an institution needed to promote women participation in the public arena).

This is clearly true – the public sphere not only needs the action of ‘policy doctors’ to diagnose and treat public problems but also ‘policy nurses’ to take care of new policies and new institutions. All of them surrounded by a polity milieu allowing for egalitarian governance frames.

In Spain, currently, we are experiencing the very dismantling of our National Health Service (NHS) (8), created under the government of the Spanish Labour party in the 1980s. The conservatives now in power (PP, partido popular) did not pass a new general health law in spite of having majority in parliament, nor are they fuelling a public debate on the privatisation of health services to abolish the existing services. They are not taking care of the institution and passing small pieces of legislation as decrees to weaken the basis of the NHS, as avoiding the care of illegal immigrants and claiming for the unsustainability of the system.

The great advantage of policy analysis comes from its ability to influence the adjacent areas of politics and polity as well. By highlighting gender bias in policies, the information is appealing to power relations and the culture, male-dominated institutions and may be leading to more equitable frames of governance.

Nevertheless, we need an enormous amount of academic work (and I recently proposed with Carmen Vives a simple, three steps – visibility and legitimation, acceptance, and true gender policies – general framework that may be useful for this task) (9). However, we will need a lot of activism to see gender equity in the near future, and I feel Belen's work is a step in the right direction.

## References

[1] Ramos M (2006). Historia de las mujeres y pensamiento feminista: una historia plural a debate. Vasconia.

[2] Oakley A (1972). Sex, gender and society: towards a new society.

[3] Wildavsky AB (1979). Speaking truth to power: the art and craft of policy analysis.

[4] Cambronero-Saiz B (2013). Gender policies and advertising and marketing practices that affect women's health. Glob Health Action.

[5] Cambronero-Saiz B, Ruiz Cantero MT, Papí-Gálvez N (2012). Quality of pharmaceutical advertising and gender bias in medical journals (1998–2008): a review of the scientific literature. Gac Sanit.

[6] Papí-Gálvez N, Cambronero-Saiz B (2011). Public actions of gender awareness. The efforts of regional and local government in advertising communication (1999–2007). PLP.

[7] Cambronero-Saiz B, Ruiz-Cantero MT, Vives-Cases C, Carrasco-Portiño M (2007). Abortion in democratic Spain: the parliamentary political agenda 1979–2004. Reprod Health Matters.

[8] Legido-Quigley H, Otero L, Parra Dl, Alvarez-Dardet C, Martin-Moreno JM, McKee M (2013). Will austerity cuts dismantle the Spanish healthcare system?. BMJ.

[9] Alvarez-Dardet C, Vives-Cases C (2012). Three waves of gender and health. Eurohealth Incorporating Euro Observer.

